# Use of volume controlled vs. pressure controlled volume guaranteed ventilation in elderly patients undergoing laparoscopic surgery with laryngeal mask airway

**DOI:** 10.1186/s12871-021-01292-y

**Published:** 2021-03-08

**Authors:** Ping Wang, Shihao Zhao, Zongbin Gao, Jun Hu, Yao Lu, Jinbao Chen

**Affiliations:** 1 Department of Anaesthesiology, Tongling People’s Hospital, 468 Bijiashan Road, Tongling, 244000 China; 2 The Second Hospital of Anhui Medical University, Hefei, China; 3 The First Affiliated Hospital of Anhui Medical University, Hefei, China

**Keywords:** Mechanical ventilation, General anesthesia, Laparoscopic surgery, Laryngeal mask airway, Elderly patients

## Abstract

**Background:**

The peak inspiratory pressure (PIP) is crucial in mechanical ventilation with supraglottic airway device (SAD). Pressure-controlled ventilation volume-guaranteed (PCV-VG), delivering a preset tidal volume with the lowest required airway pressure, is being increasingly used during general anesthesia. In this study, we compared respiratory mechanics and circulatory parameters between volume-controlled ventilation (VCV) and PCV-VG in elderly patients undergoing laparoscopic surgery using the laryngeal mask airway supreme (LMA).

**Methods:**

Eighty participants scheduled for laparoscopic surgery were enrolled in this prospective, randomized clinical trial. The participants were randomly assigned to receive VCV or PCV-VG. PIP, dynamic compliance (Cdyn) and mean inspiratory pressure (Pmean) were recorded at 5 min after induction of anesthesia (T1), 5 min after pneumoperitoneum(T2), 30 and 60 min after pneumoperitoneum (T3 and T4). Data including other respiratory variables, hemodynamic variables, and arterial blood gases were also collected. The difference in PIP between VCV and PCV-VG was assessed as the primary outcome.

**Results:**

PIP was significantly lower at T2, T3, and T4 in both groups compared with T1 (all *P* <  0.0001), and it was significantly lower in the PCV-VG group than the VCV group at T2, T3, and T4 (all *P* <  0.001). Cydn was decreased at T2, T3, and T4 in two groups compared with T1 (all *P* <  0.0001), but it was higher in PCV-VG group than in VCV group at T2, T3, and T4 (all *P* <  0.0001). There were on statistically significant differences were found between the groups for other respiratory and hemodynamic variables.

**Conclusion:**

In elderly patients who underwent laparoscopic surgery using an LMA, PCV-VG was superior to VCV in its ability to provide ventilation with lower peak inspiratory pressure and greater dynamic compliance.

## Introduction

Supraglottic airway devices (SADs) have increasingly been used in anesthesia as an effective airway maneuver [1]. Many studies have reported favorable outcomes in comfort and safety profiles [2, 3]. Meanwhile, the oropharyngeal leak pressure (OLP) is considered to be the most important determinant of the efficacy and safety of any SAD [4]. In particular, the anatomical and physiological changes that accompany ageing may influence the efficacy of a SAD in the elderly patients [5, 6]. In addition, the components of elastin fibers and collagen fibers in the hypo-epiglottic ligament is decreased with aging, making the epiglottis floppier and harder to move anteriorly [7]. Compared with young adults, the efficacy of the SAD was showed to be inferior in the geriatric population [8]. Ageing and obesity are reported to increase intraoperative ventilatory problems [9]. These problems will be more prominent in the case of elevated peak inspiratory pressure (PIP), such as the elevated airway pressure caused by pneumoperitoneum during laparoscopic surgery [10].

Pressure-controlled ventilation volume-guaranteed (PCV-VG) is an innovative mode of ventilation utilizing a decelerating flow and constant pressure. Ventilator parameters are automatically adjusted with each breath to assure the target VT without increasing inhaled airway pressures. PCV-VG has been proposed to preserve the target minute ventilation possessing advantages both of volume-controlled ventilation (VCV) and pressure-controlled ventilation (PCV) whilst producing a low incidence of barotrauma [11]. The distinctive features of the PCV-CG may lead to a decrease in PIP in the elderly patients ventilating with SAD, and we planned this prospective randomized study was to quantify the reduction in PIP during PCV-VG and VCV, and also explore the effects on respiratory and circulatory parameters in elderly patients undergoing laparoscopic surgery with laryngeal mask airway.

## Participants and methods

### Trial design and participants

This was a prospective randomized comparative clinical study which was approved by the Ethics Committee of Tongling People’s Hospital. The trial was performed in compliance with the Helsinki Declaration and data were presented in accordance with the CONSORT statement [12]. This trial was not registered in clinical trials. A total of 80 elderly participants who underwent elective laparoscopic exploration of the common bile duct between October 2017 and October 2019 and had an anticipated duration of CO_2_ pneumoperitoneum of more than 1 h were selected. Informed written consent from each participant was received. The condition of all study participants fell into the American Society of Anesthesiologists classes I and II with ages ranging from 60 to 80 years old. Exclusion criteria included those with suspected difficult intubation and severe obstructive or restrictive pulmonary diseases (defined as less than 50% of the predicted values of forced vital capacity and forced expiratory volume in 1 s) [13]. Participants who had a body mass index > 30 kg/m^2^ were not included in the study. The cohort was divided by a 1:1 ratio into either the VCV group or PCV-VG group using a computer-generated random sequence which was not blocked or stratified. The assignments were kept in sealed, opaque envelopes that were opened by an observer just before induction of anesthesia.

### Measurements, anesthesia and intervention

The primary outcome of current study is the absolute difference in PIP between VCV and PCV-VG during the first 1 h of the pneumoperitoneum. We also evaluated the differences in changes of PIP from T1 to T4 in two groups. Hemodynamic variables were measured as secondary endpoints and the other ventilatory parameters, OI, V_d_/V_T_ and were also evaluated.

No sedative premedication was given before surgery. On arrival to the operating room, standard monitoring including electrocardiography, pulse oximetry, non-invasive blood pressure and Bispectral index (BIS) monitoring were utilized. Anesthesia was induced with propofol (2 ~ 3 mg/kg), sufentanil (0.15 μg/kg). After loss of consciousness, rocuronium (0.6 mg/kg) was given and the laryngeal mask airway supreme (LMA; Teleflex Incorporated, Limerick, Maine, USA) was inserted when the nerve stimulator monitoring showed the train-of-four count was 0. LMA sizes were determined based on the participant’s body weight and manufacturers’ instructions. LMA placement was checked clinically by visual chest rise, equal bilateral alveolar sounds, and the presence of a square CO_2_ wave on capnography with manual ventilation. Following induction of general anesthesia, blood sampling and continuous blood pressure monitoring were archived via a 20G arterial catheter inserted into the radial artery of nondominant hand. After LMA insertion, all participants were machine ventilated (Dräger Perseus A500, Dräger Medical, Lubeck, Germany) and randomly assigned to the VCV or PCV-VG. The tidal volume was set to 8 mL/kg, the inspiration-to-expiration ratio (I:E) was 1:2, inspired oxygen concentration (FIO_2_) was 0.5 with air, inspiratory fresh gas flow was 2.0 L/min and no positive end-expiratory pressure (PEEP) was used throughout the operative time in both groups. End-tidal carbon dioxide (P_ET_CO_2_) was maintained between 30 and 40 mmHg by adjusting the respiratory rate (RR). Anesthesia was maintained with propofol (3 ~ 6 mg/kg/h), remifentanil (10 ~ 20 μg/kg/h) and sevoflurane (0 ~ 3%) to keep BIS values between 40 and 60. Cisatracurium besylate was continuously infused at (0.1 mg/kg/min). After the peritoneum was closed, the cisatracurium besylate infusion was stopped. Propofol and remifentanil were discontinued in all groups after wound closure. The LMA was removed when patients were able to open their eyes and the T4/T1 ratio reached 90%.Respiratory parameters, hemodynamic variables, and arterial blood gases were measured at the following time points: 5 min after induction of anesthesia and before initiation of the CO_2_ pneumoperitoneum (T1); 5 min after pneumoperitoneum (T2); 30 min after pneumoperitoneum (T3); 60 min after pneumoperitoneum (T4) [11]. The data were collected or calculated as the following: 1) Respiratory parameters: PIP, mean inspiratory pressure (Pmean), dynamic lung compliance (Cdyn), RR, Exhaled tidal volume (V_T_) and P_ET_CO_2_; 2) Arterial blood gas analysis: arterial partial pressure of oxygen (PaO_2_), arterial partial pressure of carbon dioxide (PaCO_2_); 3) Oxygenation index (OI) calculation, [PaO_2_/(FIO_2_ × Pmean)] [14]. 4) Ratio of physiologic dead space over tidal volume (V_d_/V_T_) (expressed in %) was calculated with Bohr’s formula, V_d_/V_T_ = (PaCO_2_ - P_ET_CO_2_)/PaCO_2_; 5) Hemodynamic variables: Heart rate (HR) and mean arterial pressure (MAP).

### Statistical analysis

Continuous variables were expressed as mean ± standard deviation (SD) and Kolmogorov-Smirnov test assessed the normality of the distribution of data. A two-tailed Student’s t-test determined statistical significance in the continuous data. Categorical variables were summarized as frequencies and percentages, and analyzed using χ2 or Fisher’s exact test. Variables over the study time points were analyzed using repeated-measures ANOVA with Bonferroni correction for both within-group and between-group comparisons. *P* <  0.01 was considered statistically significant. The sample size was based on previous studies [15], in which the difference in mean PIP between both modes of ventilation was 3 cm H_2_O, with a standard deviation of 3 cm H_2_O. Using α of .01 and desired power of 90%, 64 participants were determined to be needed to demonstrate a statistically significant difference. 20% more participants were recruited than necessary to compensate for drop-outs. Statistical analysis and calculations were performed using GraphPad Prism version 5.03 (GraphPad Software, San Diego, CA, USA).

## Results

**Fig. 1:**
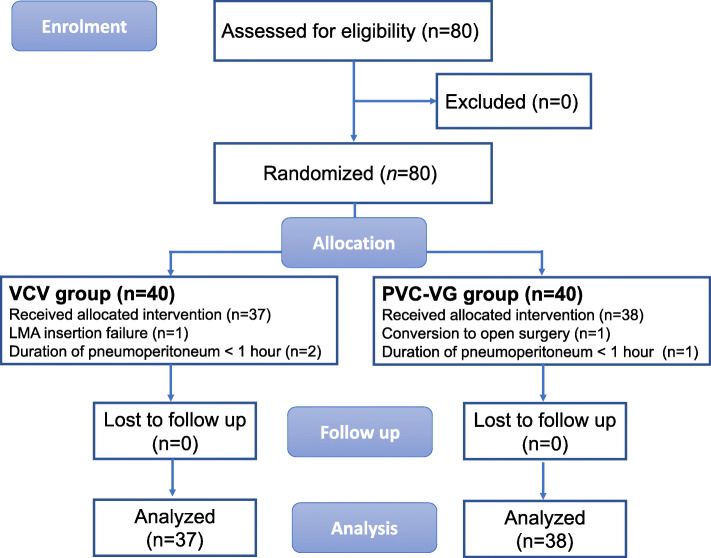
CONSORT flow diagram. VCV, volume-controlled ventilation; PCV-VG, pressure-controlled ventilation-volume guaranteed

Eighty participants were assessed for eligibility and randomized to either the VCV or PCV-VG group, and the CONSORT flow diagram was shown in Fig. 1. There were three participant’s dropouts in the VCV group, including one participant with LMA insertion failure and 2 participants with duration of pneumoperitoneum not enough than 1 h. Two participants in the PCV-VG group dropped out for one was converted to open surgery and the other involved pneumoperitoneum of less than 1 h. There were no significant differences between the groups in participant’s characteristics, preoperative pulmonary functions and operative data (Table 1).

**Table 1:** Patient characteristics, preoperative pulmonary functions and operative data

	VCV (*n* = 37)	PCV-VG (*n* = 38)
Age (year)	69.3 ± 6.4	70.6 ± 5.8
Male	18 (48.6%)	17 (44.8%)
Body Mass Index (BMI) (kg/m^2^)	20.4 ± 1.8	20.2 ± 2.3
ASA		
I	6 (16.2%)	8 (21.1%)
II	31 (83.8%)	30 (78.9%)
Preoperative FVC (% of predicted)	74.8 ± 10.9	74.5 ± 9.6
Preoperative FEV1 (% of predicted)	69.5 ± 10.0	69.0 ± 8.9
Smoking	9 (24.3%)	8 (21.0%)
Hypertension	17 (45.9%)	20 (52.6%)
Diabetes mellitus	8 (21.6%)	7 (18.4%)
First insertion success rate of LMA (%)	94.6%	94.7%
Duration of pneumoperitoneum (min)	112 ± 30	110 ± 32
Duration of surgery (min)	128 ± 29	125 ± 32

Data are presented as mean ± SD or number (percentage). *BMI* body mass index, *ASA classification* American Society of Anesthesiologists physical status classification, *FVC* forced vital capacity, *FEV1* forced expiratory volume in 1st second, *VCV* volume-controlled ventilation, *PCV-VG* pressure-controlled ventilation-volume guaranteed

**Fig. 2:**
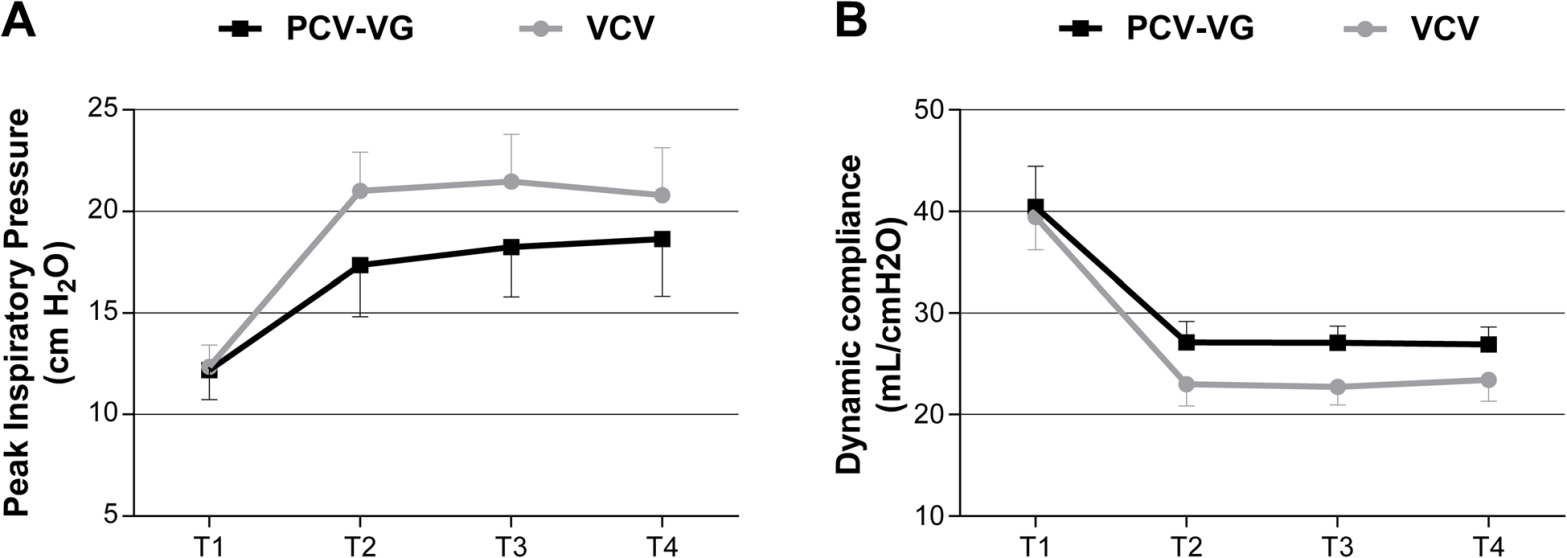
Peak Inspiratory Pressure (PIP) (**a**) and Dynamic compliance (Cdyn) (**b**) in the two groups at different stages of the study. Data are expressed as mean ± SD. T1, 5 min after induction of anesthesia and before initiation of the pneumoperitoneum; T2, 5 min after CO2 pneumoperitoneum; T3, 30 min after pneumoperitoneum; T4, 60 min after pneumoperitoneum. VCV, volume-controlled ventilation; PCV-VG, pressure-controlled ventilation-volume guaranteed

PIP was significantly higher in the VCV group than the PCV-VG group at T2 (Mean difference, 3.7; 95% CI, 2.4 to 4.9; *P* <  0.0001), T3 (Mean difference, 3.2; 95% CI, 2.0 to 4.5; *P* <  0.0001), and T4 (Mean difference, 2.2; 95% CI, 0.9 to 3.4; *P* = 0.0001). PIP was significantly increased at T2 (Mean difference, 8.6; 95% CI, 7.4 to 9.9; *P* <  0.0001), T3 (Mean difference, 9.1; 95% CI, 7.9 to 10.3; *P* <  0.0001), and T4 (Mean difference, 8.4; 95% CI, 7.2 to 9.7; *P* <  0.0001) in VCV group compared with T1, and it was also increased at T2 (Mean difference, 5.2; 95% CI, 4.0 to 6.4; *P* <  0.0001), T3 (Mean difference, 6.1; 95% CI, 4.9 to 7.3; *P* <  0.0001), and T4 (Mean difference, 6.4; 95% CI, 5.2 to 7.7; *P* <  0.0001) in PCV-VG group compared with T1 (Table 2 and Fig. 2a). Dynamic compliance was lower in VCV group than PCV-VG group at T2 (Mean difference, − 4.1; 95% CI, − 5.6 to − 2.7; *P* <  0.0001), T3 (Mean difference, − 4.3; 95% CI, − 5.8 to − 2.9; *P* <  0.0001), and T4 (Mean difference, − 3.5; 95% CI, − 4.9 to − 2.1; *P* <  0.0001). Dynamic compliance was significantly decreased at T2 (Mean difference, − 16.5; 95% CI, − 18.0 to − 14.9; *P* <  0.0001), T3 (Mean difference, − 16.7; 95% CI, − 18.3 to − 15.2; *P* <  0.0001), and T4 (Mean difference, − 16.0; 95% CI, − 17.6 to − 14.5; *P* <  0.0001) in VCV group compared with T1, and it was also lower at T2 (Mean difference, − 13.3; 95% CI, − 14.8 to − 11.8; *P* <  0.0001), T3 (Mean difference, − 13.4; 95% CI, − 14.9 to − 11.8; *P* <  0.0001), and T4 (Mean difference, − 13.5; 95% CI, − 15.1 to − 12.0; *P* <  0.0001) in PCV-VG group compared with T1 (Table 2 and Fig. 2b).

**Table 2:** Ventilatory parameters

Variables	Group	T1	T2	T3	T4	Mean diff [95% CI], *P1*	Mean diff [95% CI], *P2*	Mean diff [95% CI], *P3*
PIP (cm H_2_O)	VCV	12.4 ± 1.1	21.0 ± 1.9	21.5 ± 2.3	20.8 ± 2.3	8.6 [7.4 to 9.9], < 0.0001	9.1 [7.9 to 10.3], < 0.0001	8.4 [7.2 to 9.7], < 0.0001
	PCV-VG	12.2 ± 1.5	17.3 ± 2.5	18.3 ± 2.4	18.6 ± 2.8	5.2 [4.0 to 6.4], < 0.0001	6.1 [4.9 to 7.3], < 0.0001	6.4 [5.2 to 7.7], < 0.0001
	Mean diff [95% CI]	0.2 [1.1 to 1.4]	3.7 [2.4 to 4.9]	3.2 [2.0 to 4.5]	2.2 [0.9 to 3.4]			
	*P*	> 0.9999	< 0.0001	< 0.0001	0.0001			
Pmean (cm H_2_O)	VCV	6.2 ± 0.9	8.5 ± 1.0	8.5 ± 1.1	8.7 ± 1.0	2.4 [1.7 to 3.0], < 0.0001	2.4 [1.7 to 3.0], < 0.0001	2.5 [1.9 to 3.2], < 0.0001
	PCV-VG	6.3 ± 0.9	8.5 ± 1.2	8.5 ± 1.3	8.5 ± 1.2	2.1 [1.5 to 2.8], < 0.0001	2.1 [1.4 to 2.7], < 0.0001	2.1 [1.5 to 2.8], < 0.0001
	Mean diff [95% CI]	−0.2 [− 0.8 to 0.4]	0.1 [− 0.6 to 0.6]	0.1 [− 0.6 to 0.7]	0.2 [− 0.4 to 0.9]			
	*P*	> 0.9999	> 0.9999	> 0.9999	> 0.9999			
Cydn (ml/cm H_2_O)	VCV	39.4 ± 3.2	23.0 ± 2.1	22.7 ± 1.8	23.4 ± 2.1	−16.5 [− 18.0 to − 14.9], < 0.0001	−16.7 [− 18.3 to − 15.2], < 0.0001	− 16.0 [− 17.6 to − 14.5], < 0.0001
	PCV-VG	40.5 ± 3.9	27.1 ± 2.0	27.0 ± 1.6	26.9 ± 1.7	−13.3 [− 148 to − 11.8], < 0.0001	−13.4 [− 14.9 to − 11.8], < 0.0001	− 13.5 [− 15.1 to − 12.0], < 0.0001
	Mean diff [95% CI]	−1.0 [− 2.4 to 0.4]	−4.1 [− 5.6 to − 2.7]	−4.3 [− 5.8 to − 2.9]	−3.5 [− 4.9 to − 2.1]			
	*P*	0.3332	< 0.0001	< 0.0001	< 0.0001			
RR (breath/min)	VCV	11.9 ± 0.5	13.2 ± 0.5	14.2 ± 0.6	14.0 ± 0.6	1.3 [1.0 to 1.7], < 0.0001	2.3 [2.0 to 2.7], < 0.0001	2.2 [1.8 to 2.5], < 0.0001
	PCV-VG	11.9 ± 0.6	13.0 ± 0.5	14.1 ± 0.6	14.0 ± 0.6	1.1 [0.7 to 1.4], < 0.0001	2.1 [1.7 to 2.5], < 0.0001	1.9 [1.6 to 2.3], < 0.0001
	Mean diff [95% CI]	−0.0 [− 0.4 to 0.2]	0.2 [−0.1 to 0.5]	0.1 [− 0.2 to 0.4]	0.0 [− 0.3 to 0.4]			
	*P*	> 0.9999	0.8655	> 0.9999	> 0.9999			
V_T_ (ml)	VCV	482 ± 16	485 ± 17	480 ± 15	484 ± 18	2.0 [−8.9 to 12.8], > 0.9999	−2.5 [−13.3 to 8.4], > 0.9999	1.0 [−9.8 to 11.9], > 0.9999
	PCV-VG	483 ± 23	480 ± 22	479 ± 22	475 ± 20	−4.0 [−14.8 to 6.6], > 0.9999	−4.2 [− 14.9 to 6.5], > 0.9999	− 8.2 [− 18.9 to 2.5], 0.1961
	Mean diff [95% CI]	−0.6 [− 11.8 to 10.6]	5.4 [− 5.8 to 16.7]	1.1 [− 10.1 to 12.4]	8.7 [− 2.5 to 19.9]			
	*P*	> 0.9999	0.8965	> 0.9999	0.2117			
P_ET_CO_2_ (mmHg)	VCV	32.4 ± 2.9	33.0 ± 2.6	36.2 ± 3.2	38.7 ± 3.1	0.6 [−1.1 to 2.5], > 0.9999	3.8 [1.9 to 5.7], < 0.0001	6.3 [4.4 to 8.1], < 0.0001
	PCV-VG	32.2 ± 3.0	33.6 ± 2.9	35.5 ± 3.5	38.3 ± 4.6	1.3 [−0.5 to 3.1], 0.2646	3.2 [1.4 to 5.1], < 0.0001	6.0 [4.1 to 7.8], < 0.0001
	Mean diff [95% CI]	0.1 [−1.8 to 2.0]	− 0.5 [−2.5 to 1.4]	0.6 [− 1.2 to 2.6]	0.4 [− 1.5 to 2.3]			
	*P*	> 0.9999	> 0.9999	> 0.9999	> 0.9999			

Data are presented as mean ± SD. *PIP* peak inspiratory pressure, *Pmean* mean inspiratory pressure, *Cydn* dynamic lung compliance, *RR* respiratory rate, *P*_*ET*_*CO*_*2*_ End tidal CO2, *VCV* volume-controlled ventilation, *PCV-VG* pressure-controlled ventilation-volume guaranteed, *Mean diff* Mean difference, *CI* confidence interval

*P*, VCV vs PCV-VG; *P1*, T2 vs T1; *P2*, T3 vs T1; *P3*, T4 vs T1

In contrast, the Pmean showed no significant deference between the groups during all the time points (*P* >  0.05). P_ET_CO_2_ showed no significant deference between the two groups during all the time points yet (*P* >  0.05). There were on deference of RR at all the time points among two groups (*P* >  0.05). Both modes of ventilation ensured a stable V_T_ throughout the procedures (*P* >  0.05) (Table 2).

There were on statistically significant differences were found between the groups for PaCO_2_, PaO_2_, Oxygenation index and physiologic dead space (*P* >  0.05). (Table 3).

**Table 3:** Gas exchange values

Variables	Group	T1	T2	T3	T4	Mean diff [95% CI], *P1*	Mean diff [95% CI], *P2*	Mean diff [95% CI], *P3*
PaO_2_ (mmHg)	VCV	296 ± 30	291 ± 30	269 ± 17	266 ± 24	−5.6 [−19.2 to 7.9], 0.9539	−27.7 [−41.3 to − 14.2], < 0.0001	−30.5 [− 44.1 to − 17.0], < 0.0001
	PCV-VG	296 ± 27	297 ± 32	270 ± 17	268 ± 20	1.1 [− 12.3 to 14.5], > 0.9999	− 26.2 [− 39.5 to − 12.8], < 0.0001	−28.6 [− 41.9 to − 15.2], < 0.0001
	Mean diff [95% CI]	0.5 [− 14.2 to 15.2]	−6.2 [− 20.9 to 8.5]	− 1.1 [− 15.8 to 13.6]	−1.4 [− 16.1 to 13.3]			
	*P*	> 0.9999	> 0.9999	> 0.9999	> 0.9999			“
PaCO_2_ (mmHg)	VCV	37.8 ± 1.1	39.8 ± 2.5	42.3 ± 2.5	43.1 ± 3.2	1.9 [0.6 to 3.3], 0.0016	4.4 [3.1 to 5.8], < 0.0001	5.3 [3.9 to 6.6], < 0.0001
	PCV-VG	37.9 ± 1.0	40.0 ± 2.8	41.3 ± 2.6	42.6 ± 3.0	2.1 [0.8 to 3.4], 0.0006	3.4 [2.1 to 4.7], < 0.0001	4.7 [3.4 to 6.1], < 0.0001
	Mean diff [95% CI]	−0.0 [−1.5 to 1.4]	− 0.2 [−1.6 to 1.3]	1.0 [− 0.4 to 2.5]	0.5 [− 0.9 to 1.9]			
	*P*	> 0.9999	> 0.9999	0.2841	> 0.9999			
OI	VCV	99 ± 20	69 ± 11	64 ± 10	62 ± 10	−39.7 [−50.5 to −29.9], < 0.0001	−46.5 [− 56.2 to − 36.7], < 0.0001	− 49.6 [− 59.4 to − 39.9], < 0.0001
	PCV-VG	95 ± 17	71 ± 13	65 ± 11	64 ± 11	−32.2 [− 41.8 to − 22.6], < 0.0001	−40.4 [− 50.1 to − 30.8], < 0.0001	−41.6 [− 51.2 to − 31.9], < 0.0001
	Mean diff [95% CI]	5.0 [−5.5 to 15.4]	− 2.5 [− 12.9 to 7.9]	−1.1 [− 11.5 to 9.4]	− 3.1 [− 13.5 to 7.4]			
	*P*	0.9286	> 0.9999	> 0.9999	> 0.9999			
V_d_/V_T_ (%)	VCV	37.1 ± 2.9	39.6 ± 4.1	42.0 ± 3.7	42.3 ± 3.4	2.7 [0.7 to 4.7], 0.0036	4.8 [2.9 to 6.8], < 0.0001	5.3 [3.3 to 7.3], < 0.0001
	PCV-VG	37.1 ± 2.7	39.3 ± 4.5	40.9 ± 3.1	42.1 ± 3.9	2.2 [0.2 to 4.1], 0.0265	3.8 [1.8 to 5.7], < 0.0001	4.9 [2.9 to 6.9], < 0.0001
	Mean diff [95% CI]	−0.0 [−2.1 to 2.1]	0.5 [− 1.5 to 2.6]	1.1 [− 1.0 to 3.1]	0.3 [− 1.7 to 2.4]			
	*P*	> 0.9999	> 0.9999	0.8145	> 0.9999			

Data are presented as mean ± SD. *PaO*_*2*_ partial arterial oxygen tension, *PaCO2* partial arterial carbon dioxide tension, *OI* oxygenation index, *V*_*d*_*/Vt* Ratio of Physiologic Dead-space over tidal volume, *VCV* volume-controlled ventilation, *PCV-VG* pressure-controlled ventilation-volume guaranteed, *Mean diff* Mean difference, *CI* confidence interval

*P*, VCV vs PCV-VG; *P1*, T2 vs T1; *P2*, T3 vs T1; *P3*, T4 vs T1

Hemodynamic variables did not differ between the study groups (*P* >  0.05). However, MAP was increased at T2 (Mean difference, 7.5; 95% CI, 2.5 to 12.6; *P* = 0.0013), T3 (Mean difference, 7.8; 95% CI, 2.8 to 12.9; *P* = 0.0008), and T4 (Mean difference, 11.3; 95% CI, 6.2 to 16.4; *P* <  0.0001) compared with T1 in VCV group. And MAP was also increased in PCV-VG at T2 (Mean difference, 7.8; 95% CI, 2.8 to 12.8; *P* = 0.0007), T3 (Mean difference, 8.3; 95% CI, 3.2 to 13.3; *P* = 0.0003), and T4 (Mean difference, 9.2; 95% CI, 4.2 to 14.3; *P* <  0.0001) compared with T1. HR was stable all through the observation (*P* >  0.05) (Table 4).

**Table 4:** Hemodynamic variables

Variables	Group	T1	T2	T3	T4	Mean diff [95% CI], *P1*	Mean diff [95% CI], *P2*	Mean diff [95% CI], *P3*
MAP (mmHg)	VCV	88 ± 9	96 ± 8	96 ± 11	99 ± 8	7.5 [2.5 to 12.6], 0.0013	7.8 [2.8 to 12.9], 0.0008	11.3 [6.2 to 16.4], < 0.0001
	PCV-VG	87 ± 8	95 ± 10	95 ± 9	96 ± 8	7.8 [2.8 to 12.8], 0.0007	8.3 [3.2 to 13.3], 0.0003	9.2 [4.2 to 14.3], < 0.0001
	Mean diff [95% CI]	1.4 [−3.7 to 6.6]	1.2 [− 3.9 to 6.3]	1.0 [−4.2 to 6.1]	3.5 [−1.6 to 8.6]			
	*P*	0.9312	0.9648	0.9823	0.3084			
HR (beat/min)	VCV	78 ± 9	78 ± 11	77 ± 11	75 ± 10	0.4 [−5.8 to 6.7], > 0.9999	−1.5 [−7.8 to 4.8], > 0.9999	−2.7 [−9.0 to 3.6], 0.9166
	PCV-VG	77 ± 12	76 ± 13	77 ± 10	79 ± 11	0.9 [−7.1 to 5.3], > 0.9999	−0.3 [−6.5 to 5.9], > 0.9999	− 2.0 [−4.2 to 8.2], > 0.9999
	Mean diff [95% CI]	0.18 [− 6.2 to 6.6]	1.5 [−4.9 to 7.9]	−1.0 [− 7.5 to 5.4]	− 4.5 [− 10.9 to 1.9]			
	*P*	> 0.9999	> 0.9999	> 0.9999	0.3157			

Data are presented as mean ± SD. *MAP* mean arterial pressure, *HR* heart rate, *VCV* volume-controlled ventilation, *PCV-VG* pressure-controlled ventilation-volume guaranteed, *Mean diff* Mean difference, *CI* confidence interval

*P*, VCV vs PCV-VG; *P1*, T2 vs T1; *P2*, T3 vs T1; *P3*, T4 vs T1

## Discussion

To the best of our knowledge, this is the first prospective randomized control trial which evaluated the efficacy of PCV-VG and VCV on respiratory mechanics in elderly patients ventilated by SADs undergoing laparoscopic surgery. This study demonstrated that SAD combined with PCV-VG modal ventilation can effectively reduce PIP and improve lung dynamic compliance in elderly patients undergoing laparoscopic surgery. Such a reduction in PIP may lower the possibility of air leakage, especially when the PIP is within 3 cm H_2_O above the OLP. In such condition, conversion to endotracheal intubation is decreased. In this trial, we used the second-generation SAD, LMA supreme (LMAs; Teleflex Incorporated, Limerick, Maine, USA) which provides 26.8 cm H_2_O leak pressure [4], a high insertion success rate and ease of gastric tube insertion [16], which has led to a recent surge in popularity. The first insertion success rate was 94.59 and 94.73% in two groups which was with the most reported results [17]. The highest PIP was 26 cm H_2_O and 24 cm H_2_O in PCV-VG and VCV arms, respectively. No oropharyngeal leakages hypoxemia occurred, and the values of SpO_2_, P_ET_CO_2_, and PaO_2_ also were within normal ranges during the operation, indicating that the LMAs have an effective sealing effect and the ventilation was adequate in both groups.

The primary outcome of this study is that PCV-VG showed decreased PIP and higher dynamic compliance compared to VCV. With PCV-VG mode, the preset target tidal volume was achieved at a constant pressure through a decelerated airflow. With this system the ventilator automatically measures the respiratory mechanical parameters breath-by-breath so that each ventilation is based on the last breath. The parameters are measured in real time and affect the air flow to achieve the preset target tidal volume with the lowest airway peak pressure. PCV-VG is considered to be a time-cycled pressure adjustment mode to achieve a preset tidal volume with a variable inspiratory flow [18]. The PIP is the maximum pressure reached during the end of insufflation, it results generally from the addition of the PEEP pressure, the elastic pressure and the resistive pressure [19]. The reduction of PIP observed is probably due to the resistive pressure potentially caused by a drop in the insufflation flow rate in PCV-VG mode.

The major ventilatory doubts during laparoscopic surgery are related to the cardiopulmonary effects induced by pneumoperitoneum [20, 21]. The most prominent pulmonary change is a cephalad movement of the diaphragm, increased peak airway pressure, reduced respiratory compliance, decreased FRC and VC, and formation of atelectasis [22]. Oxygenation index may reflect atelectasis and shunt. However, oxygenation index calculated by PaO_2_/FIO_2_ is not equally sensitive on varying FiO_2_ levels such as changes in lung compliance and pulmonary shunt in mechanical ventilation settings [23, 24]. To assess the effect PCV-VG on alveolar oxygenation we measured the oxygenation index using the formulas which have been proven more accurate [14, 25, 26]. We evaluated efficiency of ventilation using V_d_/V_T_ ratio which also is the primary clinical measure. We reported the OI and V_d_/V_T_ ratio were comparable between the two modes of ventilation, and PCV-VG did not show any superiority for ventilation or oxygenation in this trial. This finding was in agreement with the results reported by Osama M et al [13] in adults underwent elective laparoscopic surgery in Trendelenburg position and a meta-analysis by Aldenkortt and colleagues [27] who also found no difference in oxygenation and ventilation with obese subjects. Contrary to our findings, Davis et al [28] suggested that PCV-VG can improve oxygenation compared with VCV in patients with acute respiratory distress syndrome. Toker MK et al reported the mean PaO_2_ levels were significantly higher in the PCV-VG group in the Trendelenburg position in obese patients [29]. In our study, the PaO_2_ were decreased slightly in both arms from T3, pneumoperitoneum can increase intrathoracic pressure [30] which results in a compression of the pulmonary capillary vessels probably and associated with increase in shunt and decrees in PaO_2_ [31]. The P_ET_CO_2_ and PaCO_2_ were increased synchronously from T3 caused by pneumoperitoneum. In order to prevent respiratory acidosis, it is necessary to intentionally increase the respiratory rate as a compromise without increasing the tidal volume. These results proposed that the patients’ ventilation had no negative effects on CO_2_ removal. As expected, in line with the increase of PaCO_2_, The MAP was also increased. However, Andersson et al. reported a distinct outcome that pneumoperitoneum causes a transient reduction of the pulmonary shunt and improved arterial oxygenation [32]. This may be explained by enhancement in HPV due to blood pressure induce by PaCO_2_ [22].

This trial also presents some limitations. First, it was not performed in a blinded fashion due to the anesthesiologist applying the noticed the mode of ventilation. Second, although this trial was based on elderly subjects, but it only included participants that were ASA 1 and 2, therefore our results only apply to relatively healthy subjects. Third, we don’t assess the efficacy of the PCV-VG on shunt and atelectasis directly, which is more significant during pneumoperitoneum. As well as we also failed to record adverse events including aspirations, laryngeal spasm, breath holding, ventilatory difficulty and postoperative respiratory complications, further research is required to evaluate the effects of PCV-VG on patient outcome.

In conclusion, in elderly patients that underwent laparoscopic surgery with PCV-VG ventilation, LMA can offer effective gas exchange and oxygenation. The major superiority of PCV-VG compared to VCV is a lower PIP and greater dynamic compliance with pneumoperitoneum.

## Data Availability

The data used and analysed during the study will be available for anyone who wishes to access them on reasonable request. The data will be accessible from immediately following publication to 6 months after publication via the first or the corresponding author by email.

## Abbreviations

SAD: Supraglottic airway devices
OLP: Oropharyngeal leak pressure
PCV-VG: Pressure-controlled ventilation volume-guaranteed
VCV: Volume-controlled ventilation
LMA: Laryngeal mask airway supreme
PIP: Peak inspiratory pressure
Cdyn: Dynamic compliance
FIO_2_: Inspired oxygen concentration
Pmean: Mean inspiratory pressure
PEEP: Positive end-expiratory pressure
V_T_: Exhaled tidal volume
P_ET_CO_2_: End-tidal carbon dioxide
BIS: Bispectral index
PaO_2_: Arterial partial pressure of oxygen
PaCO_2_: Arterial partial pressure of carbon dioxide
OI: Oxygenation index
HR: Heart rate
MAP: Mean arterial pressure
